# Previous Exercise Training Reduces Markers of Renal Oxidative Stress and Inflammation in Streptozotocin-Induced Diabetic Female Rats

**DOI:** 10.1155/2018/6170352

**Published:** 2018-03-29

**Authors:** Liliany Souza de Brito Amaral, Cláudia Silva Souza, Rildo Aparecido Volpini, Maria Heloisa Massola Shimizu, Ana Carolina de Bragança, Daniele Canale, Antonio Carlos Seguro, Terezila Machado Coimbra, Amélia Cristina Mendes de Magalhães, Telma de Jesus Soares

**Affiliations:** ^1^Programa Multicêntrico de Pós-Graduação em Ciências Fisiológicas, Instituto Multidisciplinar em Saúde, Universidade Federal da Bahia, 45029-094 Vitória da Conquista, BA, Brazil; ^2^Departamento de Nefrologia, Laboratório de Pesquisa Básica-LIM12, Faculdade de Medicina da Universidade de São Paulo, 01246-903 São Paulo, SP, Brazil; ^3^Departamento de Fisiologia, Faculdade de Medicina de Ribeirão Preto, Universidade de São Paulo, 14049-900 São Paulo, SP, Brazil

## Abstract

The aim of this study is to evaluate the effects of regular moderate exercise training initiated previously or after induction of diabetes mellitus on renal oxidative stress and inflammation in STZ-induced diabetic female rats. For this purpose, Wistar rats were divided into five groups: sedentary control (SC), trained control (TC), sedentary diabetic (SD), trained diabetic (TD), and previously trained diabetic (PTD). Only the PTD group was submitted to treadmill running for 4 weeks previously to DM induction with streptozotocin (40 mg/kg, i.v). After confirming diabetes, the PTD, TD, and TC groups were submitted to eight weeks of exercise training. At the end of the training protocol, we evaluated the following: glycosuria, body weight gain, plasma, renal and urinary levels of nitric oxide and thiobarbituric acid reactive substances, renal glutathione, and immunolocalization of lymphocytes, macrophages, and nuclear factor-kappa B (NF-*κ*B/p65) in the renal cortex. The results showed that exercise training reduced glycosuria, renal TBARS levels, and the number of immune cells in the renal tissue of the TD and PTD groups. Of note, only previous exercise increased weight gain and urinary/renal NO levels and reduced NF-*κ*B (p65) immunostaining in the renal cortex of the PTD group. In conclusion, our study shows that exercise training, especially when initiated previously to diabetes induction, promotes protective effects in diabetic kidney by reduction of renal oxidative stress and inflammation markers in female Wistar rats.

## 1. Introduction

Diabetes mellitus (DM) is a metabolic disease characterized by hyperglycemia and is associated with long-term dysfunction of various organs, especially the kidneys [1]. Although the female sex is considered a protective factor against nondiabetic kidney disease, studies suggest that in the presence of diabetes, this renoprotection is lost [2, 3]. Therefore, in order to reduce morbidity and mortality for chronic kidney disease, especially in diabetic women, it is essential to identify effective strategies to control and reduce modifiable risk factors [4]. Thus, despite the benefits of pharmacological therapies, recently, attention has turned to the inclusion of behavioral changes, including the practice of physical exercise, in order to improve metabolic control and prevent or attenuate the progression of diabetic nephropathy.

The mechanisms by which diabetes causes kidney injury are complex and not fully understood. However, the insults converge to accumulation of extracellular matrix, oxidative stress, and tissue inflammation, which are responsible for the classical alterations of diabetic nephropathy [5, 6]. In the hyperglycemic state, the autooxidation of glucose generates reactive oxygen species (ROS) accumulation with consequent reduction in the bioavailability of nitric oxide (NO) leading to oxidative damage and cytotoxicity [7]. Furthermore, reduction in the bioavailability of NO leads to other deleterious effects on the kidney such as hemodynamic changes and increased proliferative activity [8–10]. Increased ROS production can also activate proinflammatory factors such as nuclear factor kappa B (NF-*κ*B) [11]. This factor has been involved in the pathogenesis of various renal diseases [12–14], including diabetic nephropathy [15]. In addition, the infiltration of inflammatory cells, especially macrophages, is also present in the diabetic kidney and appears to contribute to the development of tissue injury [16, 17].

Regular physical activity is considered a widely accepted tool for morphological and functional protection of various target organs in chronic diseases. Studies show that exercise training prevents insulin secretion failure through the preserving *β*-cell function [18], improves metabolic control, and avoids cardiovascular autonomic dysfunction [19, 20]. Despite the overall effect of exercise on diabetic nephropathy is still controversial, recent studies have shown that exercise training of moderate intensity improves kidney function [20, 21], oxidative balance, and NO bioavailability in diabetic rats [22]. However, these studies have been focused on the exercise performed after DM induction, and so little is known about the effects of moderate exercise performed before DM induction and on the progression of diabetic nephropathy. Silva et al. were the first to demonstrate that the previous exercise training improves renal function in diabetic rats induced by streptozotocin (STZ) [20]. In a previous study, we also reported that exercise training of moderate intensity initiated before DM induction preserved renal function, reduced proteinuria, renal morphological changes, and the expression of fibronectin, type IV collagen, and TGF-*β* in renal cortex in STZ-induced diabetic female rats [23]. However, the mechanisms by which the previous exercise mediated these renal benefits are not clear. Therefore, considering that oxidative stress and inflammation are essential processes in the pathogenesis of diabetic nephropathy, we aimed to compare the effects of moderate intensity exercise training initiated prior of exercise training of moderate intensity started prior or after the induction of DM on the oxidative stress and inflammation in STZ-induced diabetic female rats.

## 2. Materials and Methods

All experimental procedures were approved by the Ethics Committee on Animal Experimentation of the Instituto Multidisciplinar em Saúde, Universidade Federal da Bahia, (protocol 008/2013) and were strictly conducted according to the recommendations of the National Institutes of Health Guide for the Care and Use of Laboratory Animals.

### 2.1. Animals and Experimental Protocol

Female Wistar rats, weighing 180–200 g, were housed under controlled environmental conditions (24 ± 2°C; 12/12 h light/dark cycle) with free ad libitum access to water and rodent chow. The rats were divided into the following groups: sedentary control (SC, *n* = 6); trained control (TC, *n* = 6); sedentary diabetic (SD, *n* = 7); trained diabetic (TD, *n* = 6), and previously trained diabetic (PTD, *n* = 7). Only the PTD group was submitted to four weeks of aerobic training before DM induction, while the other groups remained sedentary. After these four weeks, DM was induced in the SD, TD, and PTD groups. One week after the induction of DM, the TC, TD, and PTD groups were submitted to the aerobic physical exercise protocol for eight weeks. We measured body weight weekly and collected blood and 24-hour urine samples for thiobarbituric acid reactive substances (TBARS), nitric oxide (NO), and glycosuria dosages. After that, we submitted all animals to decapitation and collected blood and kidneys for TBARS, glutathione (GSH), NO assays, and immunohistochemical studies.

### 2.2. Diabetes Mellitus Induction

After 12 h fasting, animals weighing 290–310 g were made diabetic by a single intravenous injection of STZ (Alfa Aesar, Ward Hill, MA, USA) (40 mg/kg, i.v.) diluted in 0.1 M citrate buffer (pH 4.5). Control animals were injected with citrate buffer alone. One week after STZ injection, DM was confirmed by measurement of capillary glycemia with Accu-Chek glucose strips (Roche, Mannheim, Germany). The rats with capillary glycemia higher than 250 mg/dL were considered diabetics.

### 2.3. Exercise Training Protocol

We submitted only the PTD group to four weeks of aerobic exercise training on the treadmill before inducing DM. The previous exercise was started with 20 min of treadmill running in the first week and increased up to 1 h by the end of the fourth week. Similarly, exercise intensity was also progressively increased (55–70%) building up to 70% toward the end of the protocol [20, 23]. This protocol was performed with no inclination of the treadmill, during five consecutive days per week, which is considered as an exercise of moderate intensity for diabetic rats [19–23]. One week after DM induction, the TC, TD, and PTD groups were subjected to the same training protocol described above over a period of 8 weeks.

### 2.4. Glycosuria

Glycosuria was analyzed by colorimetric method (Abbott Diagnostics Kit) using an automatic biochemical analyzer (Abbott Diagnostics C.4100, Saint-Laurent, Quebec, Canada).

### 2.5. Tissue Sample Collection and Preparation

After euthanasia, the right kidneys were frozen in liquid nitrogen and stored at −80°C to determine TBARS, NO, and glutathione levels. Tissue samples were homogenized in ice-cold isolation solution (200 mM manitol, 80 mM HEPES, and 41 mM KOH, pH 7.5) containing a protease inhibitor cocktail (Sigma Chemical Company, St. Louis, MO, USA). The homogenates were centrifuged for 15 min at 4°C to remove nuclei and cell debris. After that, we isolated the supernatants and quantified the protein amount by Bradford assay method (Bio-Rad Protein Assay kit, Bio-Rad Laboratories, Hercules, CA, USA). The left kidneys were also removed and weighed. Then, we cut a small slice of the renal tissue and fixed in methacarn solution for 24 hands in 70% alcohol thereafter. Kidney blocks were embedded in paraffin and cut into 4 *μ*m sections for immunohistochemical studies.

### 2.6. Plasma, Urinary, and Renal TBARS Levels

Plasma, urinary, and renal levels of thiobarbituric acid reactive substances (TBARS) were determined using the thiobarbituric acid assay [24]. In brief, a 0.2 mL plasma or urinary sample was diluted in 0.8 mL of distilled water. Immediately, 1 mL of 17.5% trichloroacetic acid was added. Following the addition of 1 mL of 0.6% thiobarbituric acid, pH 2, the sample was placed in a boiling water bath for 15 min, after which it was allowed to cool. Subsequently, 1 mL of 70% trichloroacetic acid was added, and the mixture was incubated for 20 min. The sample was then centrifuged for 15 min at 2000 rpm. The optical density of the supernatant was read at 534 nm against a reagent blank using a spectrophotometer. The quantity of TBARS was calculated using a molar extinction coefficient of 1.56 × 105 mol^−1^/cm^−1^. The TBARS levels in renal tissue were corrected by the amount of proteins (mg) contained in the sample and expressed in nmol/mg, whereas the levels of urinary TBARS were corrected by urinary volume of 24 h and expressed as nmol/24 h.

### 2.7. Renal Glutathione Levels

Reduced glutathione (GSH), the major endogenous antioxidant in cells, was determined in tissue homogenate according to Sedlak and Lindsay [25]. Renal tissue homogenate was processed by the addition of four volumes of ice-cold 5% metaphosphoric acid and centrifuged at 4000 rpm for 10 min at 4°C. This assay consists of the action of supernatants with Ellman's reagent to produce a yellow pigment measured spectrophotometrically at 412 nm. GSH was quantified by means of the standard curve and reported as *μ*mol/mL/mg protein.

### 2.8. Indirect Determination of Serum, Urinary, and Renal Tissue NO Levels

NO levels were estimated through the nitrate levels present in the serum, urine, and renal tissue samples. Total nitrate was measured using the Sievers Nitric Oxide Analyzer System. The samples were deproteinized and submitted to a reaction containing vanadium trichloride (VCl3), which converts nitrate to NO. The NO produced was detected by ozone induced by chemiluminescence. Peak values of NO samples were determined using a standard curve constructed with sodium nitrate solutions of various concentrations (5, 10, 25, 50, 100, and 1000 *μ*M). The NO levels in renal tissue were corrected by the amount of proteins (mg) contained in 1000 *μ*L of the homogenate and expressed in *μ*mol/mg, whereas urinary levels of NO were corrected by urinary volume of 24 h and expressed as *μ*mol/mL/24 h.

### 2.9. Antibodies

We performed our immunohistochemical studies with the following primary antibodies: a monoclonal anti-ED1 antibody (AbD Serotec, Oxford, UK), which reacts with a single-chain glycoprotein with a molecular weight of 110 kDa, that is expressed on the lysosomal membranes of macrophages and monocytes; a monoclonal anti-CD43 antibody (Abcam, Cambridge, UK), which reacts with a heavily glycosylated glycoprotein with a molecular weight of 95 kDa, that is expressed on T lymphocytes; and a polyclonal anti-NF-*κ*B (p65) antibody (Abcam, Cambridge, UK). In unstimulated cells, NF-*κ*B is bound to canonical inhibitors I*κ*B*α* and I*κ*B*β* and found in cytoplasm. When these cells are stimulated, the release of NF-*κ*B from canonical inhibitors results in the passage of p65 and p50 subunits into the nucleus, where it binds to specific sequences in the promoter regions of target genes involved in many biological processes such as inflammation, differentiation, cell growth, and apoptosis [11].

### 2.10. Immunohistochemistry Studies

Histological sections of renal tissue were incubated for 1 hour at room temperature with monoclonal anti-ED1 antibody (1 : 300) and overnight at 4°C with the antibodies anti-CD43 monoclonal (1 : 200) and anti-NF-*κ*B p65 polyclonal (1 : 100) [13]. The reaction product was detected with avidin-biotin-peroxidase complex (Vector Laboratories, Burlingame, CA, USA), and the color reaction was developed with 3,3-diaminobenzidine (Vector Laboratories, Burlingame, CA, USA). The sections were then counterstained with methyl green or hematoxylin. Negative controls consisted of replacing the primary antibodies with equivalent concentrations of normal rabbit IgG. Except for ED-1, nonspecific protein binding was blocked by incubation with 20% goat serum in PBS for 30 min. We evaluated the immunostaining for NF-*κ*B (p65) using ImageJ software, and the results were expressed as percentages, while the quantification for ED-1 and CD43 was performed by counting positive cells in 30 fields (0.245 mm^2^) of the tubulointerstitial compartment of each histological slide.

### 2.11. Statistical Analysis

All quantitative data were expressed as mean ± SEM. Differences among the means of multiple parameters were analyzed by one-way analysis of variance followed by the Student-Newman-Keuls test. Analysis of correlations was performed by Pearson's test. Values of *p* < 0.05 were considered statistically significant. Data were analyzed using GraphPad Prism software 5.0 (La Jolla, CA).

## 3. Results

### 3.1. Body Weight Gain and Glycosuria

We observed a reduction in body weight gain in all diabetic groups after ten weeks of DM induction. However, this alteration was lower in the PTD group compared to the SD group (*p* < 0.05) (Table 1). Diabetic groups presented urinary glucose excretion. However, the TD and PTD groups showed a reduction in glycosuria compared to the SD group (*p* < 0.01) (Table 1).

### 3.2. TBARS and Glutathione Levels

We noticed higher levels of TBARS in the kidney of the SD group compared to the control groups (*p* < 0.01). Interestingly, exercise attenuated such levels in the TD and PTD groups when compared to the SD group (*p* < 0.05). There were no differences in serum/urinary TBARS and tissue glutathione levels among the experimental groups (Table 2).

### 3.3. NO Levels in Serum, Urine, and Kidney

**Figure 1:**
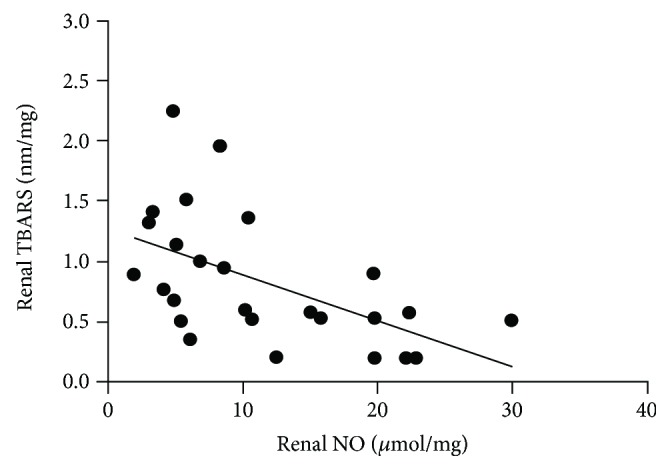
Correlation between TBARS and NO renal levels of SC, TC, SD, TD, and PTD rats. *n* = 5 for all groups. Pearson's correlation, *r* = −0.54, *p* < 0.01.

The SD and TD groups showed a reduction in NO tissue levels compared to the control groups (*p* < 0.05). Of note, previous exercise prevented this alteration (*p* < 0.05). Urinary excretion of NO was also lower in the SD group (*p* < 0.05), and the previous exercise reversed this effect in the PTD group (*p* < 0.05) (Table 2). NO levels were negatively correlated with TBARS levels in kidney homogenates (*r* = −0.54, *p* < 0.01) (Figure 1).

### 3.4. ED-1, CD43, and NF-*κ*B (p65) Immunostaining

**Figure 2:**
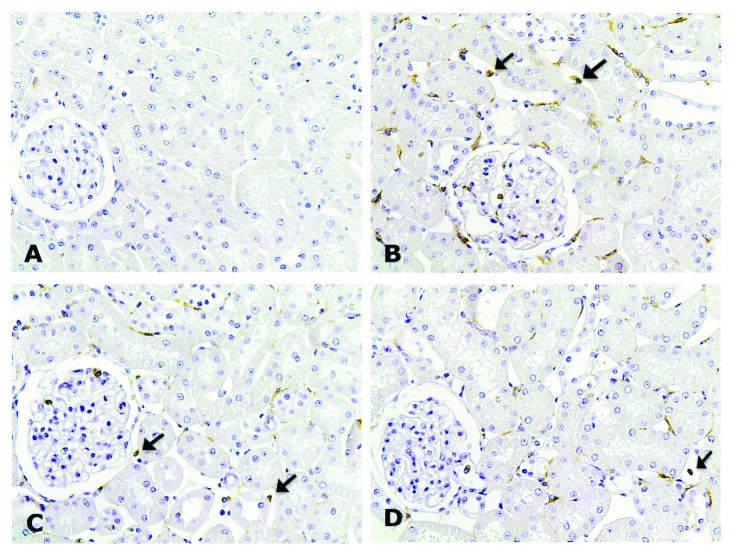
Immunolocalization of CD43 in the renal cortex of SC (a), SD (b), TD (c), and PTD (d) rats. Note the less intensity of CD43 immunostainings in PTD. Original magnification ×200.

**Figure 3:**
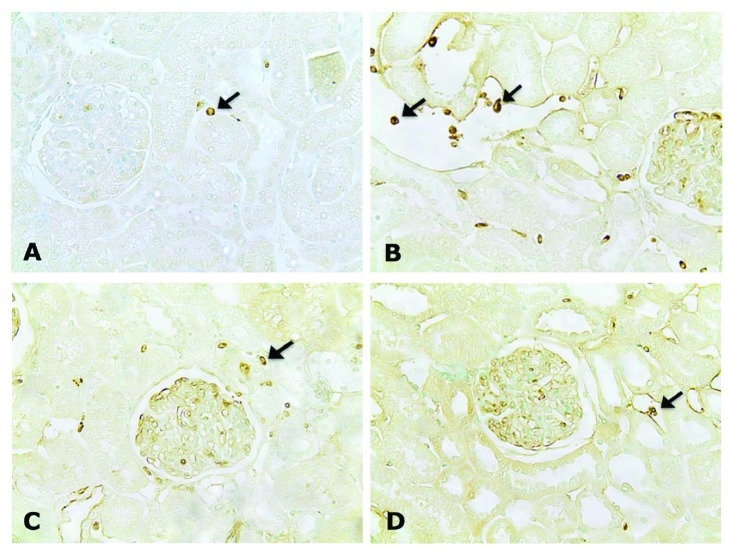
Immunolocalization of ED-1 in the renal cortex of SC (a), SD (b), TD (c), and PTD (d) rats. Note the less intensity of ED-1 immunostainings in PTD. Original magnification ×200.

**Figure 4:**
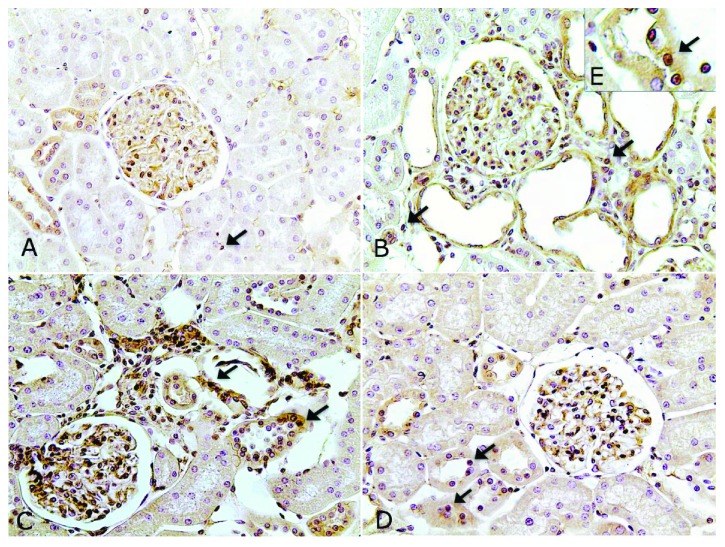
Immunolocalization of NF-*κ*B (p65) in the renal cortex of SC (a), SD (b), TD (c), and PTD (d) rats. Note the nuclear immunostaining of NF-*κ*B in (e), suggesting the migration of this factor to the nucleus. Note also lesser intensity of NF-*κ*B immunostaining in PTD. Original magnification ×200.

The SD group showed an increased number of CD43-positive cells (*p* < 0.001) in the cortical tubulointerstitial compartment compared to the control groups. Importantly, exercise reduced the number of these cells in the TD and PTD groups (*p* < 0.01) compared to the SD group (Table 3) (Figure 2). Only the SD group showed an increase in the number of ED-1-positive cells compared to the controls (*p* < 0.05), while exercise prevented this effect in the trained diabetic groups (Table 3) (Figure 3). The SD and TD groups showed a more intense immunostaining for NF-*κ*B (p65) in the tubulointerstitial compartment of the renal cortex when compared to the control groups. As a highlight, only the previous exercise attenuated this effect in the PTD group when compared to the SD and TD groups (*p* < 0.01) (Table 3) (Figure 4). Microphotographs of the TC group were not presented due to the similarity with the SC group.

## 4. Discussion

In a previous study from our laboratory, we reported that moderate intensity exercise training initiated before DM induction improved metabolic control and renal function and reduced proteinuria, renal morphological changes, and expression of extracellular matrix proteins in renal cortex in STZ-induced diabetic female rats [23]. In the present study, we investigated the mechanisms by which exercise promotes these benefits to the diabetic kidney, and we suggest that the reduction of oxidative stress and inflammation markers can contribute to this process. Our data demonstrate that both exercise protocols were able to confer protection to diabetic kidney through improved metabolic control and reduced renal levels of TBARS and inflammatory cells. More important, additional benefits were achieved only when the exercise was started previously to DM induction, such as increased renal levels of NO and reduced NF-*κ*B expression.

In our study, both exercise training reduced glycosuria of the TD and PTD groups compared to the SD group. In addition, improved metabolic benefits were achieved by previous exercise, since only the PTD group showed increase in weight gain. These results suggest that previously trained animals presented a reduction of the catabolic process, which may have contributed for the attenuation of renal changes investigated in this study. Silva et al. also showed that exercise training started prior to DM induction was more effective in improving metabolic capacity than exercise performed only after the onset of the disease [20]. However, other studies have shown that exercise started after the DM establishment was also able to improve glycemic control and reduce weight loss in STZ-induced diabetic rats [19, 22].

High levels of TBARS in renal tissue observed in the SD group are a strong indication that oxidative stress may have contributed to the development of tissue lesions and renal function changes, which we have demonstrated in our previous study in the same experimental model [23]. Oxidative stress is a major mechanism involved in the progression of diabetic nephropathy. Chronic hyperglycemia leads to increased ROS production, which contributes to lipid peroxidation of cell membranes and formation of lipid products, especially malondialdehyde (MDA) [26]. MDA is a highly cytotoxic TBARS product capable to cause additional damage cell membrane, inflammation, and tissue necrosis [27, 28]. In addition, superoxide anion can interact with NO to form peroxynitrite, a highly cytotoxic radical, leading to a reduction in the bioavailability of NO [28]. In this study, besides the increase in the renal TBARS levels, the SD group also showed a reduction in tissue and urinary levels of NO, suggesting that a reduction of the bioavailability of this gas may also have contributed to the increased renal injury in these animals.

The molecular mechanisms by which aerobic exercise provides protective action on diabetic kidney are not fully elucidated. However, there is evidence indicating that the redox state improvement and the increases of production and bioavailability of NO may be involved in this process [22, 29, 30]. Thus, Rodrigues et al. showed that 8 weeks of moderate aerobic exercise reduced the urinary and renal TBARS and increased the urinary and renal NO levels in STZ-induced diabetic rats [22]. In another experimental model of renal injury induced by NO deficiency, aerobic exercise increased the SOD activity and the nitrite/nitrate serum ratio, besides to reduce the renal TBARS levels [30]. Moreover, D'ischia et al. demonstrated that under aerobic conditions, an increased production of exercise-induced NO could also inhibit the formation of TBARS through decomposition of lipid peroxidation products [31]. Thus, such evidences are consistent with the findings of this study since, in fact, both training protocols have reduced TBARS renal levels in the TD and PTD groups. However, it is important to note that only the previous exercise increased the NO levels in the PTD group.

There were no changes in renal glutathione levels among experimental groups, probably because the imbalance of redox state at this stage of DM has not been sufficient to reduce the antioxidant defenses. However, we observed a negative correlation between TBARS and NO renal levels, suggesting that higher TBARS levels in the SD group may have contributed to the reduction of NO bioavailability in these animals. On the other hand, reduced TBARS renal levels in the PTD group may have contributed to the attenuation of renal lesions and conservation of NO physiological levels promoted by previous exercise.

In addition to oxidative stress, the subclinical and low-grade inflammation is also involved in the pathogenesis of diabetic nephropathy [32]. Although the structural changes induced by diabetes primarily affect the glomeruli, the tubulointerstitial lesions elicit an inflammatory response in the tubulointerstitial compartment, with infiltration of mononuclear cells, which is strongly correlated with disease progression [17]. Thus, our data show an increase in the number of ED-1and CD43-positive cells and higher NF-*κ*B immunostaining in the renal cortex of the SD group. The participation of the NF-*κ*B has been shown in several experimental models of renal disease [13, 33, 34]. In diabetic nephropathy, Tone et al. demonstrated that NF-*κ*B activation resulted in macrophage infiltration in the renal tubulointerstitial compartment and production of proinflammatory cytokines in renal tissue of diabetic rats [35].

Several studies have shown that the interstitial infiltration of lymphocytes and macrophages contribute to the aggravation of renal tubulointerstitial injury by generation of ROS and stimulation of proinflammatory cytokines, including TNF-*α* [13, 36–38]. The increase of ROS and TNF-*α*, in addition to causing tissue damage, also contributes to the activation of NF-*κ*B, thereby generating a vicious cycle able to perpetuate itself and contribute to the progressive loss of renal function [13, 39, 40]. Similarly, in our study, increased oxidative stress may have contributed to the activation of NF-*κ*B and increased numbers of inflammatory cells in the renal cortex of the SD group. On the other hand, exercise training was able to attenuate renal inflammatory process in the trained diabetic groups, since the number of CD43-positive cells was reduced in these animals compared to the SD group. Moreover, increased NF-*κ*B immunostaining in the renal cortex of the SD group rats was completely reversed by previous exercise in the PTD group. So far, there are no studies demonstrating the effects of previous exercise on renal inflammation in diabetic nephropathy. However, it has been shown that a reduced formation of ROS and peroxynitrite induced by regular aerobic exercise was associated with reductions in TNF-*α* expression and NF-*κ*B activity in the kidney of spontaneously hypertensive rats [29]. Asghar et al. also demonstrated that lower MDA levels and higher SOD levels promoted by regular aerobic exercise were accompanied by attenuation of C-reactive protein levels and increased IL-10 levels in aged rats [41]. Therefore, based on this evidence, we can suppose that the decreases of oxidative stress and renal inflammatory process may be related in PTD animals.

## 5. Conclusion

In conclusion, our data suggest that regular moderate intensity exercise training, particularly when initiated prior to the emergence of DM, exerts protective action on diabetic kidney by reducing renal markers of oxidative stress and inflammation and increasing bioavailability of NO, thus slowing the progression of diabetic nephropathy in STZ-induced diabetic female rats.

## Figures and Tables

**Table 1:** Body weight gain and glycosuria of SC, TC, SD, TD, and PTD rats.

	SC	TC	SD	TD	PTD
ΔBW (%)	84.15 ± 12.7	64.40 ± 4.9	−4.507 ± 3.3^∗∗∗^	2.816 ± 8.9^∗∗∗^	28.67 ± 12.8^∗^^#^
Glycosuria (mg/24 h)	*Undetected*	*Undetected*	2481 ± 328.5	1301 ± 119.5^##^	1192 ± 99.61^##^

Data are expressed as mean ± standard error. ΔBW: body weight gain. ^∗∗∗^*p* < 0.001 and ^∗^*p* < 0.05 versus SC and TC; ^##^*p* < 0.01 and ^#^*p* < 0.05 versus SD. The ΔBW was calculated by the equation [(final weight − initial weight)/initial weight] × 100.

**Table 2:** TBARS and NO levels in serum, urine, and kidney and GSH levels in the kidney of SC, TC, SD, TD, and PTD rats.

	SC	TC	SD	TD	PTD
*Renal tissue*					
TBARS (nm/mg)	0.38 ± 0.08	0.39 ± 0.08	1.96 ± 0.45^∗∗^	0.86 ± 0.15^#^	1.26 ± 0.09^#^
NO (*μ*mol/mg)	15.11 ± 2.85	22.13 ± 2.31	9.09 ± 1.72^∗^	6.34 ± 1.11^∗^	19.09 ± 2.88^#††^
GSH (*μ*mol/mg)	0.35 ± 0.04	0.31 ± 0.04	0.40 ± 0.08	0.28 ± 0.04	0.25 ± 0.05
*Serum*					
TBARS (nm/mL)	1.29 ± 0.2	1.38 ± 0.12	1.15 ± 0.11	1.33 ± 0.28	1.75 ± 0.18
NO (*μ*mol/mL)	71.92 ± 25.52	137.5 ± 26.73	86.60 ± 15.50	111.5 ± 17.09	102.3 ± 21.23
*Urine*					
TBARS (nm/24 h)	186.9 ± 21.09	118.3 ± 21.15	217.5 ± 70.02	231.4 ± 58.36	252.3 ± 42.99
NO (*μ*mol/24 h)	12.09 ± 2.32	14.73 ± 1.44	5.18 ± 1.97^∗^	8.56 ± 2.72	15.52 ± 3.49^#^

Data are expressed as mean ± standard error. TBARS_S_, TBARS_U_, and TBARS_R_; thiobarbituric acid reactive substance serum (nm/mL), urinary (nm/24 h), and renal tissue (nm/mg), respectively; GSH_R_: renal glutathione (*μ*mol/mg); NO_S_, NO_U_, and NO_R_; nitric oxide serum (*μ*mol/mL), urinary (*μ*mol/24 h), and renal tissue (*μ*mol/mg), respectively. ^∗∗^*p* < 0.01 and ^∗^*p* < 0.05 versus SC and TC; ^#^*p* < 0.05 versus SD; ^††^*p* < 0.01 versus TD.

**Table 3:** Renal immunostaining for ED-1, CD43, and NF-*κ*B (p65) of SC, TC, SD, TD, and PTD rats.

	SC	TC	SD	TD	PTD
ED-1	3.9 ± 0.2	3.6 ± 0.2	6.9 ± 0.9^∗^	6.3 ± 0.6	4.8 ± 1.0
CD43	*Undetected*	*Undetected*	7.0 ± 0.5	2.8 ± 0.8^##^	1.7 ± 0.7^##^
NF-*κ*B	0.5 ± 0.3	0.9 ± 0.3	4.7 ± 0.9^∗^	6.1 ± 1.4^∗∗^	1.3 ± 0.6^##††^

Data are expressed as mean ± standard error. ED-1: macrophage marker; CD43: lymphocyte marker; and NF-*κ*B: nuclear factor kappa B. ^∗∗^*p* < 0.01 and ^∗^*p* < 0.05 versus SC and TC; ^##^*p* < 0.01 versus SD; ^††^*p* < 0.01 versus TD.
